# Total Body Irradiation in Haematopoietic Stem Cell Transplantation: A Comprehensive Literature Review and Institutional Experience from the Policlinico of Catania

**DOI:** 10.3390/medicina61091503

**Published:** 2025-08-22

**Authors:** Maria Chiara Lo Greco, Roberto Milazzotto, Grazia Acquaviva, Rocco Luca Emanuele Liardo, Giorgia Marano, Madalina La Rocca, Antonio Basile, Pietro Valerio Foti, Stefano Palmucci, Emanuele David, Corrado Iní, Lorenzo Aliotta, Vincenzo Salamone, Viviana Anna La Monaca, Stefano Pergolizzi, Corrado Spatola

**Affiliations:** 1 Radiation Oncology Unit, Department of Biomedical, Dental and Morphological and Functional Imaging Sciences, University of Messina, 98122 Messina, Italy; mariachiaralg@gmail.com (M.C.L.G.); giorgiamarano@gmail.com (G.M.); madalina.larocca@gmail.com (M.L.R.); stefano.pergolizzi@unime.it (S.P.); 2 Radiation Oncology Unit, University Hospital Policlinico “G. Rodolico-San Marco”, 95123 Catania, Italy; gra.acquaviva@libero.it (G.A.); lucaliardo@hotmail.com (R.L.E.L.); 3 Department of Medical Surgical Sciences and Advanced Technologies “G.F. Ingrassia”, University of Catania, 95123 Catania, Italy; basile.antonello73@gmail.com (A.B.); pietrofoti@hotmail.com (P.V.F.); spalmucci@unict.it (S.P.); david.emanuele@yahoo.it (E.D.); corrado.ini@gmail.com (C.I.); lore.aliotta@gmail.com (L.A.); 4 Radiology I Unit, University Hospital Policlinico “G. Rodolico-San Marco”, 95123 Catania, Italy; 5 U.O.S.D. Fisica Sanitaria, University Hospital Policlinico “G. Rodolico-San Marco”, 95123 Catania, Italy; v.salamone@policlinico.unict.it (V.S.); v.lamonaca@policlinico.unict.it (V.A.L.M.)

**Keywords:** TBI, HSCT, TMI, TMLI

## Abstract

*Background and Objectives*: Total body irradiation (TBI) remains a cornerstone of conditioning for allogeneic haematopoietic stem-cell transplantation (HSCT). Whereas early research debated the need for irradiation, contemporary investigations focus on optimising dose, fractionation and delivery techniques. *Material and Methods*: We synthesised six decades of evidence, spanning from single-fraction cobalt treatments to modern helical tomotherapy and intensity-modulated total-marrow/lymphoid irradiation (TMI/TMLI). To complement the literature, we reported our institutional experience on 77 paediatric and adult recipients treated with conventional extended-source-to-skin-distance TBI at the University Hospital Policlinico “G. Rodolico–San Marco” between 2015 and 2025. *Results*: According to literature data, fractionated myeloablative schedules, typically 12 Gy in 6 fractions, provide superior overall survival and lower rates of severe graft-versus-host disease (GVHD) compared with historical single-dose regimens. Conversely, reduced-intensity protocols of 2–4 Gy broaden HSCT eligibility for older or comorbid patients with acceptable toxicity. Conformal planning reliably decreases mean lung dose without compromising engraftment, and early-phase trials are testing selective escalation to 16–20 Gy or omission of TBI in molecularly favourable cases. With regard to our institutional retrospective series, 92% of patients completed a 12-Gy regimen with only transient grade 1–2 nausea, fatigue or hypotension; all transplanted patients engrafted, and no grade ≥ 3 radiation pneumonitis occurred. *Conclusions*: Collectively, the published evidence and our experience support TBI as an irreplaceable component of HSCT conditioning and suggest that coupling it with advanced imaging, organ-sparing dosimetry and molecular response monitoring can deliver safer, more personalised therapy in the coming decade.

## 1. Introduction

Despite significant advancements in chemotherapy-based conditioning regimens for haematological malignancies, Total Body Irradiation (TBI) has remained, over recent decades, a cornerstone for haematopoietic stem cell transplantation (HSCT), particularly in clinical situations where penetration of radiation in sanctuary organs confers advantages over systemic therapy alone [1].

The foundations of modern HSCT date back to the 1950s, when E. Donnall Thomas and colleagues first combined TBI with bone marrow infusion, a pioneering approach that ultimately earned a Nobel Prize and established the basis for contemporary bone marrow transplantation [2].

Early TBI regimens employed single-fraction radiation using cobalt machines, which posed several challenges, including complex patient positioning and notable side effects [3,4]. The introduction of novel technologies led to the development of linear accelerators and refined fractionation schedules, markedly improving delivery time and treatment tolerability [5,6]. Today, TBI continues to evolve, with the emergence of reduced-intensity regimens and targeted approaches like total marrow irradiation (TMI) and total marrow and lymphoid irradiation (TMLI), which aim to preserve efficacy while minimising toxicity [7].

This work aims to present a comprehensive overview of the evolution of TBI, with a particular focus on fractionation schedules, delivery techniques, and ongoing challenges. To this end, we performed a PubMed search on TBI and HSCT, prioritising randomised trials, prospective cohort studies, and comparative analyses. In parallel, we report over one decade of institutional experience at the University Hospital Policlinico of Catania, to highlight the development and adaptation of local protocols in response to technological advancements and shifting clinical indications.

## 2. TBI Indications and Integration with Systemic Therapy

TBI is a specialised radiotherapy technique that delivers a uniform dose of ionising radiation to the entire body [4]. Initially used as a standalone treatment, TBI has since become an integral part of conditioning regimens for HSCT for different haematological malignancies, with both myeloablative and immunosuppressive intents [8,9].

In allogeneic HSCT, especially for acute leukaemia, TBI facilitates the eradication of residual haematopoietic cells and helps create immunologic space for donor cell engraftment [10].

Historically, one of the earliest and most widely used radiotherapy schedules for TBI was a single 10 Gy fraction. However, since the early 1980s, fractionated TBI has demonstrated superior outcomes, including improved overall survival and lower rates of graft-versus-host disease (GVHD) [11,12].

Nowadays, the typical myeloablative TBI regimens include:

- 12 Gy delivered as 2 Gy twice daily over 3 days (6 fractions) [13];
- 12–13.5 Gy delivered as 1.5 Gy twice daily over 4–4.5 days (8–9 fractions) [13];
- 12–13.2 Gy delivered as 1.2 Gy three times daily over 4 days (10–11 fractions) [13];
- 12 Gy delivered as 3 Gy once daily over 4 days (4 fractions) [13];

In the paediatric population, the typical myeloablative doses range between 12 and 14.4 Gy (1.6–2 Gy per fraction) [14]. Additionally, in acute lymphoblastic leukaemia, a testicular boost of 4 Gy (2 Gy × 2 fractions) is often administered to reduce relapse in this sanctuary site, although this practice varies by institution and is under continued evaluation [15].

Regarding chemotherapy regimens, the most widely adopted agent is cyclophosphamide; however, high-dose etoposide can be considered an alternative for high-risk or refractory cases where deeper cytoreduction is needed [16].

In efforts to replace TBI in the conditioning of acute leukaemia, several studies have investigated non-TBI containing regimens, including intravenous busulfan and cyclophosphamide [17,18]. Despite these efforts, TBI has demonstrated to improve event-free survival rates as well as overall survival and is therefore still a key component for allogeneic HSCT. In particular, the superiority of fractionated TBI over chemotherapy-only conditioning has been confirmed by the phase-III FORUM trial, which significantly improved the 2-year overall survival rates (0.91; 95% CI, 0.86 to 0.95; *p* < 0.0001 versus 0.75; 95% CI, 0.67 to 0.81) and cumulative incidence of relapse (0.12; 95% CI, 0.08 to 0.17; *p* < 0.0001 versus 0.33; 95% CI, 0.25 to 0.40) in paediatric and adolescents and young adults (AYA) affected by acute lymphoblastic leukaemia [19].

For elderly patients or those with comorbidities, reduced-intensity conditioning (RIC) regimens are increasingly adopted, with total doses ranging from 1 to 8 Gy [13]. The Quesenberry group demonstrated that even 1 Gy of TBI could already support engraftment in a series of 25 patients with refractory cancer [20,21]. Similarly, the Seattle group showed that 2 Gy TBI with fludarabine is sufficient to achieve reliable engraftment in 45 patients with haematologic malignancies, ineligible for conventional HSCT [22].

Nowadays, RIC regimens of 2 Gy TBI, alone or in association with fludarabine or fludarabine and melphalan, offer sufficient immunosuppression for full donor chimerism while minimising regimen-related toxicity [23,24]. In cases requiring intensified immunosuppression, such as with HLA-mismatched donors or heavily pretreated recipients, the TBI dose may be escalated to 4 Gy (2 Gy × 2 fractions) [25].

The development of RIC regimens, including thiotepa-based conditioning, has allowed for the expansion of transplant eligibility over the years [26]. However, the potential risks of graft rejection or disease relapse must still be considered.

In the autologous HSCT setting, particularly for multiple myeloma, relapsed Hodgkin lymphoma, and non-Hodgkin lymphoma, TBI was historically incorporated into conditioning regimens due to its cytoreductive properties [9]. However, its role has diminished over time and is now largely reserved for selected high-risk cases.

An overview of fractionation schedules based on their indications is reported in Table 1.

## 3. Technological Advancements in TBI

### 3.1. From Isotopes to Linear Accelerators

Some of the earliest experiences reported in the literature on TBI date back to the late 1950s, when multiple radiation sources were used to achieve adequate dose distribution.

In 1961, Jacobs and Pape described one of the first dedicated TBI chambers based on cesium-137 sources. The sources were contained in rods placed at the extremity of the treatment bed, at 2 m, and were controlled by lead filters and by an on/off system [27].

From cesium-137 to cobalt-60, in 1959, Sahler developed one of the first dual-source cobalt-based radiation units for TBI, which allowed the delivery of a parallel opposed field at about 3 m from the patient, with a low dose rate irradiation (<10 cGy/min) [28].

The following innovation utilised a single cobalt-60 source and was reported in 1981 by investigators from the Princess Margaret Hospital in Toronto. At that time in the facility, the radiation unit was engineered to deliver a large radiation field at a relatively short source-to-skin distance (SSD) of 90 cm, with high dose rates (100–200 rad/min). Despite the challenges of dosimetry for such a large field, this advance represented a practical and high-output solution for that time [29].

Subsequently, several innovations were made, including vertical-beam TBI and translation-irradiation technique, until the implementation of photon beams linear accelerators, paving the way for conventional methods [30,31,32]

Conventional TBI refers to a specific delivery approach where the patient lies or stands at an extended SSD of around 3–4 m and is treated with a single radiation beam and with the largest possible field size. With the aim of obtaining a uniform dose rate along the patient’s body, compensating filters or boluses are adopted, together with a Plexiglas plate, usually placed between the radiation source and the patient’s body, to counteract the skin-sparing effect. Regarding positioning verifications, a light field simulating the irradiation field is normally used. Anterior and posterior fields are adopted, with the dose prescribed to the mid-plane [33].

To reduce the dose to the lungs, organ shielding is performed through customised blocks of high-density material, accurately positioned and verified with the use of radiographic films [34]. However, attention should be paid to reach an adequate beam transmission, balancing the risk of interstitial pneumonia and of leukaemia recurrence. Nowadays, Computed Tomography (CT) simulation-based solutions are adopted for accurate shielding in a minimal treatment time [35].

### 3.2. Intensity Modulated Total Body Irradiation

Starting from the early 2000s, the most recent technological advancements in radiotherapy have enabled the delivery of highly conformal radiation to even large anatomical volumes. Techniques such as intensity-modulated radiotherapy (IMRT), volumetric modulated arc therapy (VMAT), Helical Tomotherapy (HT), and Image-Guided Radiotherapy (IGRT) allow for more precise targeting of the bone marrow, lymphatic structures, and circulating blood, while offering improved results in distribution. These innovations have led to the development of TMI and TMLI, which are organ-sparing approaches that refine traditional TBI by selectively irradiating haematopoietic and lymphoid tissue [36].

Early experiences with TMI with HT already showed the technical feasibility, dosimetric precision, and clinical benefits of this approach over traditional methods, although associated with longer treatment planning and delivery times [37].

Compared to conventional TBI, VMAT and HT allow for a more comfortable patient position and better sparing of organs at risk with fewer acute toxicities. However, the dual treatment plan requires meticulous planning to avoid over- and under-dosing in the junctions; with an overall treatment duration that can extend up to 30–90 min, due to the necessity to irradiate the entire body in sequential sections [38,39]

Overall, excellent results have been obtained with conventional linear accelerators, although highlighting the necessity to develop novel planning techniques to account for large treatment fields, as well as the integration of daily cone-beam CT to verify patient position [40].

Nowadays, TMI and TMLI are normally delivered through static or dynamic HT or VMAT and involve a meticulous patient setup, immobilisation, and imaging [41,42,43].

In the simulation phase, the patient lies in the supine position and is normally immobilised with the help of a thermoplastic mask and a personalised vacuum pillow. Based on the patient’s size, two separate CT scans (head-first and feet-first) and a dual treatment plan might be necessary to cover the entire body of the patient [41]. To facilitate changing positions, a rotating couch top is often required.

Unlike conventional TBI, an accurate definition of the clinical target volume (CTV) is needed, including the skeletal bone, the entire lymphatic system when indicated, as well as other sanctuary sites such as the testicles and the brain [41]. CTV is then expanded to the planning target volume (PTV), with a margin that is usually based on the institutional protocol.

Organs at risk typically include critical structures such as the brain (if not part of the target volume), eyes, lenses, optic nerves and chiasm, oral cavity, major salivary glands, thyroid, oesophagus, lungs, heart, breasts, stomach, liver, spleen (if excluded from the target), kidneys, small and large intestines, rectum, bladder, and genital organs [41].

Despite these benefits, the most advanced irradiation techniques have not demonstrated superiority, compared to conventional TBI, in terms of immunosuppressive effect on bone marrow cells and tumoricidal effect on circulating leukaemic cells. Furthermore, no substantial difference between modern techniques and conventional TBI has been shown in the occurrence of relapses [44].

## 4. Ongoing Challenges and Future Directions

### 4.1. Cellular Hallmarks: Key Considerations

As early as the 1990s, experiments conducted on leukaemic cell cultures revealed a heterogeneous response to radiation, with some cell lines exhibiting marked radiosensitivity and others demonstrating significant radioresistance [45,46].

Building on this work, Wheldon discovered that a TBI regimen of 7 fractions of 2 Gy could result in a wide spectrum of treatment responses in leukaemic cells, ranging from easily curable to entirely unresponsive cases [46].

Although the mechanisms driving this variability were not fully understood then, and still today remain incompletely defined, early work referred to dysregulation of apoptosis-related genes (bcl2, p53, c-myc) and cellular differentiation pathways as possible key contributors [47,48,49].

In a more recent study, for example, Li et al. demonstrated that activation of the Hedgehog signalling pathway mediates resistance to radiation in acute myeloid leukaemia, with cell lines exhibiting enhanced DNA repair and suppressed apoptosis. Importantly, the pharmacologic inhibition of this pathway with LDE225 restored radiosensitivity both in vitro and in vivo, providing proof-of-concept that molecular targeting can be used to reverse resistance and personalise TBI strategies [49,50].

Besides genetic alteration, differences in radiobiological parameters were also reported as potential contributors to radiation response heterogeneity, namely the α/β ratio [46,50]. The α/β ratio defines the fraction dose where both α (non-reparable DNA damage) and β (reparable DNA damage) components cause the same value of cell death, and is therefore higher in early responding tissues (less sensitive to fraction size or dose rate variations), while is lower in late-responding tissues (more susceptible to fraction size or dose rate increments) [51]. Cosset et al. reported high variability in cell survival curves within haematological malignancies, with low fraction size sensitivity for acute non-lymphoblastic leukaemia, but observed significant fractionation sensitivity in chronic myeloid leukaemia, along with notable interindividual variability in acute lymphoblastic leukaemia [52].

Collectively, these genetic and radiobiological considerations paint a picture of highly individual radiation sensitivity. To translate that bench-to-bedside decision-making, modern molecular response tools have also recently been developed to identify individualised metrics. Specifically, advanced techniques such as PCR-based minimal residual disease (MRD) monitoring and next-generation sequencing (NGS) are increasingly used to assess patient-specific tumour burden and treatment response [53,54].

Furthermore, early-phase clinical trials have successfully incorporated radiolabelled anti-CD20, -CD45, and -CD66 antibodies into standard TBI-based conditioning regimens for HSCT [55,56,57]. These approaches represent a major step toward Wheldon’s vision of biologically targeted, spatially precise irradiation, enhancing tumoricidal effect where needed while potentially reducing systemic toxicity.

### 4.2. Ongoing Clinical Trials

Over the past decades, several prospective studies have confirmed TBI’s role as part of conditioning for allogeneic HSCT. In parallel, the introduction of haploidentical HSCT has broadened donor availability and led to the evaluation of TBI-containing regimens also in this setting [10,58]. Accordingly, research focus has shifted from whether to irradiate to how best to do so, with a special focus on reducing long-term sequelae, especially in paediatric and AYA survivors (endocrine and metabolic disfunctions, neurocognitive effects, secondary malignancies, cataracts, etc.) [14].

Treatment-modality optimisation is now being investigated by a multi-institutional phase-I study (NCT04281199), which is testing whether highly conformal IMRT-TBI (12 Gy in 6 fractions) can keep the mean lung dose below 8 Gy while still covering ≥ 85% of the target volume. If successful, it will provide the first prospective evidence that modern IMRT can cut the historical pulmonary cost of myeloablative TBI without losing antileukaemic potency [56].

Complementing this dosimetric approach, a phase-II escalation trial at City of Hope (NCT04262843) is pushing IMRT TMI/TMLI to 20 Gy while enforcing the same < 8 Gy lung- and kidney-dose threshold, seeking to determine whether selectively intensifying marrow/lymphoid dose can further improve disease control in high-risk acute myeloid leukaemia/myelodysplastic syndrome [57].

Dose de-escalation is being pursued in two parallel “mini-TBI” studies that give only 4 Gy in two fractions: City of Hope combines low-dose TBI with fludarabine-melphalan for haplo-identical grafts (NCT03118492), while Roswell Park adds post-transplant cyclophosphamide to curb GVHD (NCT03192397) [58,59].

Biology-guided omission of irradiation is under investigation in the phase-II EndRAD trial (NCT03509961), which assigns children, adolescents, and young adults with B-cell acute lymphoblastic leukaemia who are negative for minimal residual disease as shown by next-generation sequencing to a fully myeloablative, radiation-free conditioning regimen [60]. Patients who remain positive for minimal residual disease continue on the conventional observational arm that delivers 12 Gy total-body irradiation in 6 fractions. The study will clarify whether these excellent-risk patients can forego total-body irradiation and its long-term pulmonary, endocrine, and neuro-cognitive complications without sacrificing disease control [60].

At the opposite end of the spectrum, dose-intensification strategies are also emerging. The single-arm phase-II studies by the University of Chicago (NCT06802315, NCT04187105) are testing 9–6 Gy IM-TMI boosts within fludarabine–busulfan or fludarabine–cyclophosphamide–based conditioning to high-risk acute myeloid leukaemia/myelodysplastic syndrome or haplo-identical recipients, respectively, aiming to expand transplant eligibility while capping critical-organ doses [61,62].

In parallel, a first-in-human phase I/II study at Fred Hutchinson combines an ultra-low-dose (≤2 Gy) TBI with an astatine-211-labelled anti-CD45 monoclonal antibody (NCT04083183) to test “α-conditioning” as a highly targeted, organ-sparing alternative for non-malignant indications [63].

Ongoing trials are listed in Table 2.

Taken together, these complementary trials, spanning conformal delivery, de-escalation, complete omission and selective intensification, promise to delineate a data-driven therapeutic window in which irradiation can be tailored to each patient’s disease biology and tolerance, thereby maximising cure while minimising late toxicities.

## 5. Experience at the University Hospital Policlinico G. Rodolico San Marco

To give a complementary clinical insight on local protocols and their development in response to technological advancements, here we report over one decade of institutional experience at the University Hospital Policlinico “G. Rodolico-S. Marco” of Catania.

Between January 2015 and July 2025, we delivered conventional TBI to 77 patients, including both adults and paediatric patients (median age 28 years old). The underlying diagnoses were B- or T-cell acute lymphoblastic leukaemia (*n* = 52), acute myeloid leukaemia (*n* = 5), Hodgkin or non-Hodgkin lymphoma (*n* = 13), aplastic anaemia (*n* = 3), mycosis fungoides (*n* = 3), and treatment-refractory chronic myeloid leukaemia (*n* = 1).

Most patients (*n* = 51) received an immunosuppressive schedule of 12 Gy in 2 Gy fractions, administered twice daily with a 4–6 h interfraction interval. Four patients followed an intermediate regimen of 8 Gy (2 Gy × 2 daily) combined with intensified systemic therapy, while 17 patients, mainly with relapsed Hodgkin or non-Hodgkin lymphoma, aplastic anaemia, or mycosis fungoides, underwent single-fraction 2 Gy.

**Figure 1:**
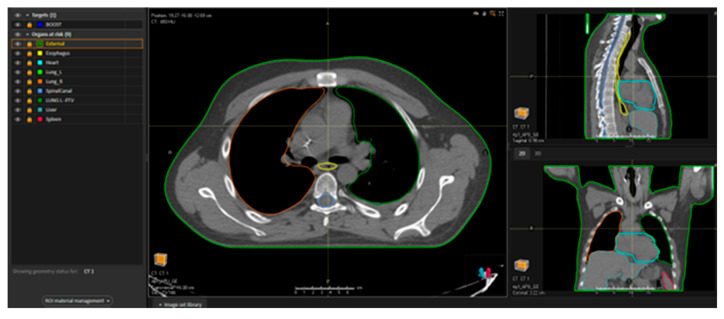
Simulation CT scan (axial, sagittal, and coronal axes) visualised on our institutional treatment planning system.

**Figure 2:**
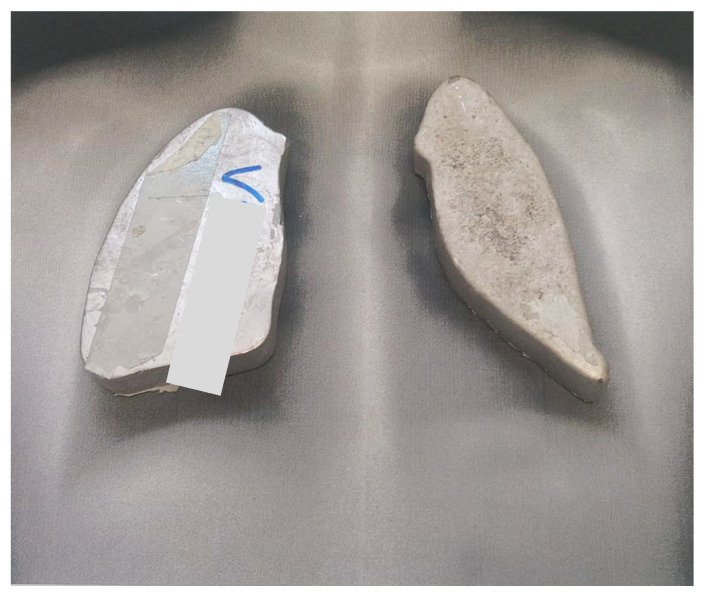
Individual blocks for lung shielding.

For all 8–12 Gy courses, a non-contrast chest simulation CT scan was performed (Figure 1) to quantify lung density and to enable fabrication of individual blocks (Figure 2) that limited pulmonary exposure to ≈8–10 Gy.

**Figure 3:**
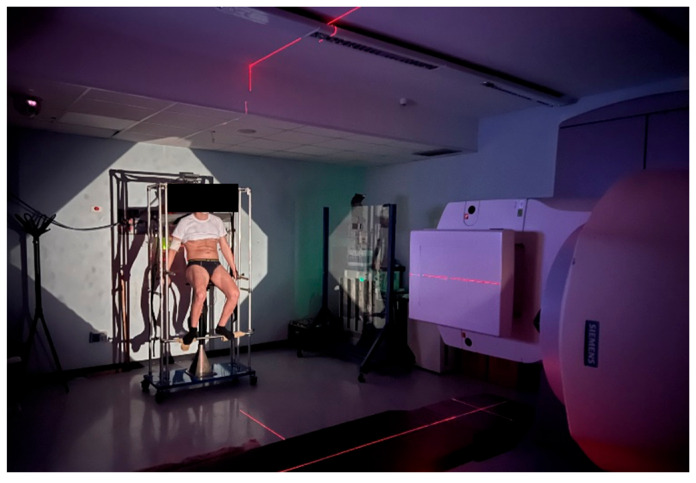
Visual representation of a conventional TBI treatment session.

Treatment was delivered in a semi-standing position with the gantry at 90° and the collimator at 45°, placing the whole body along the field diagonally (Figure 3).

**Figure 4:**
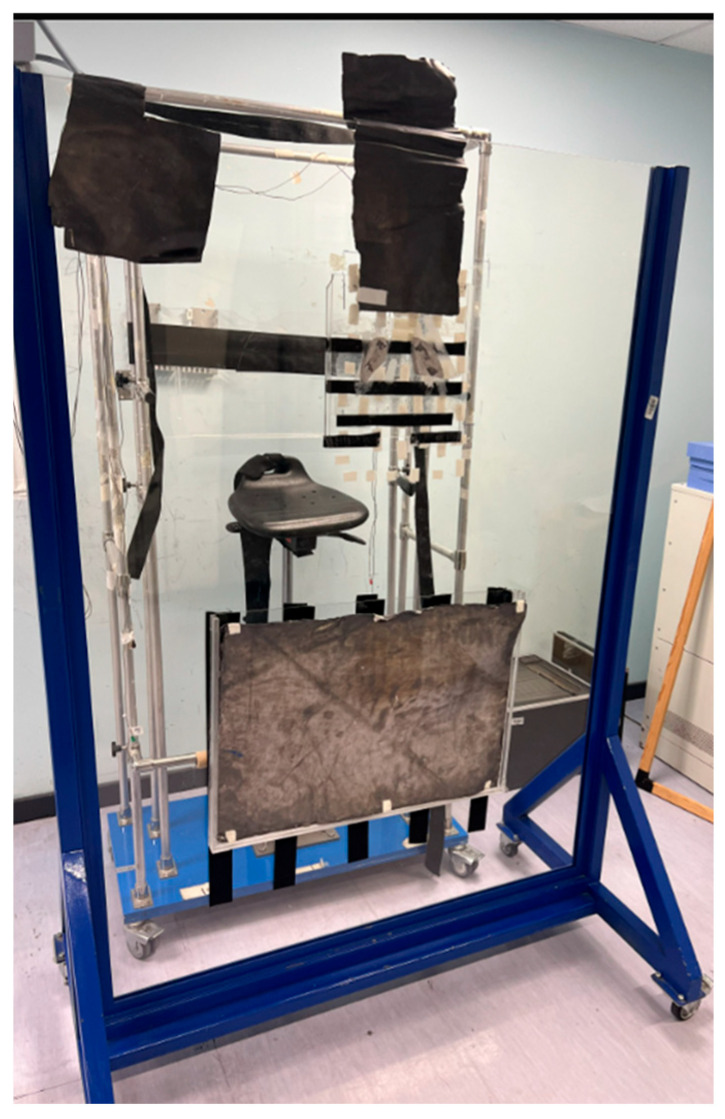
Plexiglas spoiler for skin-sparing effect.

**Figure 5:**
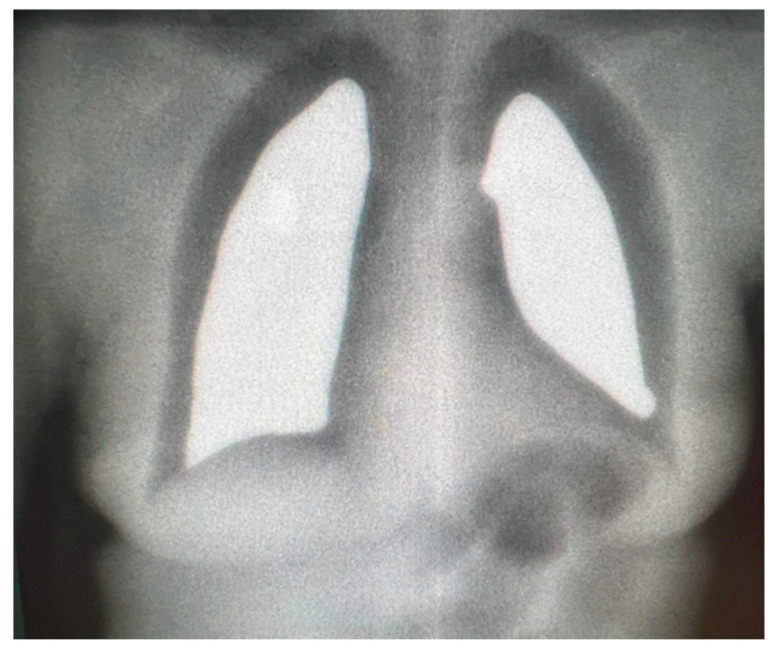
Lung shielding placement verification.

A Plexiglas spoiler countered the skin-sparing effect of 6 MV photons (Figure 4). Alternating AP and PA fields ensured dose homogeneity; this was verified each fraction by in vivo dosimetry; orthogonal X-rays confirmed block placement before irradiation (Figure 5). All patients received anti-emetic and corticosteroid pre-medication.

Despite the demanding schedule, 71 of 77 patients (92%) completed TBI, and 68/77 (95.8%) proceeded with allogeneic haematopoietic cell transplantation. Of these, 18/68 patients (26.5%) received haploidentical grafts. Acute toxicities were monitored during the entire treatment course and included nausea and vomiting, syncope, and severe fatigue in up to 80% of patients. Daily full blood counts were used to monitor leucopenia and neutropenia.

Although long-term toxicity data was not available at the time of analysis, results confirm the feasibility of conventional TBI in the context of HSCT, highlighting, however, the need for optimal interdisciplinary collaboration and appropriate patient monitoring and support.

To keep pace with recent technological advances, our centre is now developing intensity-modulated protocols for TBI and will possibly start including TMI and TMLI in clinical practice.

## 6. Conclusions

Over seven decades, total body irradiation has evolved from single-shot cobalt treatments to sophisticated intensity-modulated platforms, yet its biological rationale, such as uniform myeloablation and potent immunosuppression, remains unchanged. The literature consistently shows that fractionated myeloablative schedules (e.g., 12 Gy in 6 fractions) improve survival and reduce GVHD with historical single-dose regimens, while reduced-intensity protocols (2–4 Gy) safely extend transplant eligibility to older or medically fragile patients. Parallel advances in treatment delivery, including VMAT, HT, and emerging total-marrow/lymphoid irradiation, now allow centimetre-level conformality and organ sparing without compromising engraftment or disease control.

Our 10-year series of 77 paediatric and adult recipients treated with conventional extended-SSD TBI corroborates these broader observations. A 92% completion rate, acceptable acute toxicity, and successful engraftment in nearly all eligible patients affirm the robustness of traditional techniques even in the era of precision radiotherapy. Nonetheless, the dosimetric discipline required—individual lung blocks, in vivo dosimetry, and labour-intensive setup—highlights a pressing need to streamline workflows. Commissioning of IMRT-based TMI or TMLI at our centre is therefore a logical next step, promising shorter sessions, lower pulmonary doses, and a platform for future biologically guided dose painting.

Looking ahead, the convergence of molecular disease monitoring, and targeted radionuclide conjugates foreshadows a shift from “one-size-fits-all” irradiation to truly personalised conditioning. Ongoing phase-I/II trials testing conformal 12 Gy regimens, 4 Gy de-escalation, and MRD-stratified omission of TBI will clarify how best to balance efficacy against long-term sequelae. Multi-institutional cooperation and prospective data collection—including late toxicity, quality-of-life, and health-economic endpoints—will be essential to translate these innovations into new standards of care.

In summary, total body irradiation continues to be an indispensable, versatile component of haematopoietic stem-cell transplantation. By marrying the proven curative potential of TBI with modern imaging, dosimetry, and biologic insight, the field is poised to deliver safer, more effective, and more patient-tailored conditioning in the decade to come.

## Figures and Tables

**Table 1:** Summary of fractionation schedules per conditioning regime and corresponding indications.

Schedule	Administration	Conditioning	Clinical Indications	Key Notes
10 Gy/1 fraction [11,12]	QD over 1 day	Historical myeloablative	Early AML/ALL transplants (pre-1980)	Excess toxicity; largely obsolete
12 Gy/6 fractions [13]	BID over 3 days	Standard myeloablative	Front-line paediatric and adult allo-HSCT	Benchmark OS & GVHD profile
12–13.5 Gy/8–9 fractions [13]	BID over 4–4.5 days	Standard myeloablative	Centres favouring smaller fraction sizes	Slightly longer course
12–13.2 Gy/10–11 fractions [13]	TID over 4 days	Hyper-fractionated myeloablative	Centres favouring smaller fraction sizes	Lowest per-fraction dose
12 Gy/4 fractions [13]	QD over 4 days	Condensed myeloablative	Logistical constraints or BID-intolerant patients	Requires meticulous lung shielding
4 Gy testicular boost/2 fractions [14]	QD over 2 days	Sanctuary boost	Boys with ALL	Practice varies; omission under study
1 Gy/1 fraction [19,20]	QD over 1 day	Minimal-intensity/experimental	Older/comorbid adults;	Very low toxicity
2 Gy/1 fraction [21]	QD over 1 day	Standard RIC	Older/comorbid adults	Very low toxicity
4 Gy/2 fractions [24]	BID or QD over 1–2 days	Augmented RIC	Mismatched donors or high-risk disease	Balances better engraftment vs. added toxicity

QD = once daily; BID = twice daily; TID = three times daily; RIC = reduced-intensity conditioning; AML = acute myeloid leukaemia; ALL = acute lymphoblastic leukaemia; HSCT = haematopoietic stem-cell transplantation; OS = overall survival; GVHD = graft-versus-host disease.

**Table 2:** Ongoing trials details.

Trial	Aim	Investigational Arm	Status
NCT04281199 (Phase I) [56]	IMRT-TBI dosimetric optimisation	Conformal IMRT-TBI 12 Gy/6 fx	Active, not recruiting
NCT04262843 (Phase II) [57]	Escalated TMI/TMLI to 20 Gy	TMLI 20 Gy (lung < 8 Gy)	Recruiting at the main site, but several satellites are now “Active-not-recruiting”
NCT03118492 (Phase I) [58]	4 Gy mini-TBI + Flu-Mel in haplo HSCT	4 Gy (2 × 2 Gy) + Flu-Mel	Active, not-recruiting
NCT03192397 (Phase Ib/II) [59]	4 Gy mini-TBI + PTCy	4 Gy (2 × 2 Gy) + PTCy	Active, not recruiting
NCT03509961 (EndRAD, Phase II) [60]	MRD-guided omission of TBI	Chemo vs. 12 Gy TBI	Recruiting
NCT06802315 (BMT-13, Phase II) [61]	High-risk AML/CML/MDS	IM-TMI 9 Gy + Flu-Bu	Active, not recruiting
NCT04187105 (BMT-06, Phase II) [62]	Haplo-HSCT dose-escalated TMI	IM-TMI 6 Gy + Flu-Cy + PTCy	Recruiting
NCT04083183 (Phase I/II) [63]	^211^At-anti-CD45 α-conditioning	≤2 Gy TBI + ^211^At-BC8-B10	Recruiting

AML = acute myeloid leukaemia; At = astatine-211; α-conditioning = targeted astatine-211–anti-CD45 alpha-particle conditioning; Bu = busulfan; CML = chronic myeloid leukaemia; Cy = cyclophosphamide; Flu = fludarabine; fx = fraction (s); Gy = grey; HSCT = haematopoietic stem-cell transplantation; IM-TMI = intensity-modulated total marrow irradiation; IMRT = intensity-modulated radiotherapy; MDS = myelodysplastic syndrome; Mel = melphalan; MRD = measurable residual disease; PTCy = post-transplant cyclophosphamide; TBI = total body irradiation; TMI = total marrow irradiation; TMLI = total marrow and lymphoid irradiation.

## Data Availability

The data presented in this study are available on request from the corresponding author. The data are not publicly available due to privacy restrictions.
